# Cytocidal Antitumor Effects against Human Ovarian Cancer Cells Induced by B-Lactam Steroid Alkylators with Targeted Activity against Poly (ADP-Ribose) Polymerase (PARP) Enzymes in a Cell-Free Assay

**DOI:** 10.3390/biomedicines9081028

**Published:** 2021-08-17

**Authors:** Nikolaos Nikoleousakos, Panagiotis Dalezis, Aikaterini Polonifi, Elena G. Geromichalou, Sofia Sagredou, Constantinos E. Alifieris, Maria V. Deligiorgi, Vasiliki Sarli, Dimitrios T. Trafalis

**Affiliations:** 1 Laboratory of Pharmacology, Faculty of Medicine, National and Kapodistrian University of Athens, 11527 Athens, Greece; pdalezis@med.uoa.gr (P.D.); kater_pol@yahoo.com (A.P.); elena_geromich@outlook.com (E.G.G.); ssagredou@med.uoa.gr (S.S.); kalifier@med.uoa.gr (C.E.A.); mdeligiorgi@yahoo.com (M.V.D.); dtrafal@med.uoa.gr (D.T.T.); 2 Department of Chemistry, Aristotle University of Thessaloniki, University Campus, 54124 Thessaloniki, Greece; sarli@chem.auth.gr

**Keywords:** anticancer drug, B-lactam steroid alkylators, synthetic lethality, poly (ADP-ribose) polymerase inhibitors, ovarian cancer, hybrid steroidal alkylating agents

## Abstract

We evaluated three newly synthesized B-lactam hybrid homo-aza-steroidal alkylators (ASA-A, ASA-B and ASA-C) for their PARP1/2 inhibition activity and their DNA damaging effect against human ovarian carcinoma cells. These agents are conjugated with an alkylating component (POPA), which also served as a reference molecule (positive control), and were tested against four human ovarian cell lines in vitro (UWB1.289 + BRCA1, UWB1.289, SKOV-3 and OVCAR-3). The studied compounds were thereafter compared to 3-AB, a known PARP inhibitor, as well as to Olaparib, a standard third-generation PARP inhibitor, on a PARP assay investigating their inhibitory potential. Finally, a PARP1 and PARP2 mRNA expression analysis by qRT-PCR was produced in order to measure the absolute and the relative gene expression (in mRNA transcripts) between treated and untreated cells. All the investigated hybrid steroid alkylators and POPA decreased in vitro cell growth differentially, according to the sensitivity and different gene characteristics of each cell line, while ASA-A and ASA-B presented the most significant anticancer activity. Both these compounds induced PARP1/2 enzyme inhibition, DNA damage (alkylation) and upregulation of PARP mRNA expression, for all tested cell lines. However, ASA-C underperformed on average in the above tasks, while the compound ASA-B induced synthetic lethality effects on the ovarian cancer cells. Nevertheless, the overall outcome, leading to a drug-like potential, provides strong evidence toward further evaluation.

## 1. Introduction

The second most common malignancy after breast cancer in women over the age of 40 is ovarian cancer. As of 2018, ovarian cancer was the seventh most common cancer worldwide in women and the fifth leading cause of cancer-related death in the same group. Due to the lack of a definitive screening tool and the vague signs and symptoms that can “masquerade” as other non-malignant conditions, curative and survival trends have not changed significantly, as early diagnosis remains a challenge [1].

Currently, the most common treatment consists of surgery and a combination of platinum/taxane chemotherapy [2]. Nevertheless, the treatment is often ineffective, since a major percentage of patients acquire resistance to chemotherapy regimens. The Platinum-resistant disease constricts further known chemotherapy approaches; treatment protocols at this point are often selected individually, but recurrent disease is typically not curable [3].

Inherited mutations in the BRCA1 and BRCA2 genes and the Lynch syndrome (hereditary nonpolyposis colorectal cancer) have been closely associated with a highly increased risk of developing ovarian cancer [4]. The protein products of the aforementioned genes and their functions have been extensively investigated, in reference to the repair of DNA strand breaks via homologous recombination [5] and the detriment of this ability (referred to as “BRCAness”) [6]. This “malfunction” renders the cell susceptible to further DNA alterations and increases the dependency on PARP-mediated DNA repair [7]. 

Poly (ADP-ribose) polymerase (PARP) is a superfamily of 18 proteins characterized by the PARP homology domain, the catalytic domain. This catalytic domain helps in the ADP-ribosylation of various acceptor proteins using nicotinamide adenine dinucleotide (NAD+) as a donor for ADP-ribose. PARP-1 and PARP-2 carry out 80% of the poly-ADP-ribosylation of cellular protein [8]. Poly-ADP-ribosylation of nuclear proteins, a post-translational event, occurs in response to DNA damage. Poly-(ADP-ribose) polymerase (PARP) catalyzes the NAD-dependent addition of poly-(ADP-ribose) to itself and adjacent nuclear proteins such as histones. The contribution of PARP to the events that occur during DNA base excision repair (BER) is important [9].

Nevertheless, PARP overactivation depletes its substrate, NAD+, slowing the rate of glycolysis, electron transport and ATP formation, eventually leading to the functional cell impairment or a PARP-mediated induction of cell and tissue necrosis by extensive depletion of the intracellular NAD pool [10,11]. The cleavage of PARP-1 promotes apoptosis by preventing DNA repair-induced survival and by blocking energy depletion-induced necrosis [12].

Moreover, PARP activation promotes the transcription of pro-inflammatory genes and modulates important inflammatory pathways, while PARP-1 transcription and activity can be modulated as well by several endogenous mechanisms, such as “suicide” enzymes, like caspases and hormonal factors, including steroid hormones. Consequently, as has been presented in various studies, PARP inhibition could lead to several therapeutic outcomes by downregulating multiple simultaneous pathways of inflammation and tissue injury, including beneficial effects on neuronal and myocardial ischemia/reperfusion injury, various forms of heart failure, cardiomyopathies, circulatory shock, cardiovascular aging, diabetes and diabetic cardiovascular complications, myocardial hypertrophy, atherosclerosis, vascular remodeling following injury, angiogenesis of myocardial ischemia, septic shock, vascular stroke, arthritis, colitis and neurodegenerative disorders [11,13,14,15].

The pharmacological effects of PARP inhibitors potentially enhance the cytotoxicity of certain DNA-damaging anticancer drugs and promote the chemosensitization and radiosensitization of tumors [15,16]. 

The aim of this report was to assess the potential antitumor effects of three novel B-lactam-steroid alkylating compounds on human ovarian cancer cells, together with their potential to inhibit PARP1/PARP2 activity.

The tested hybrid homo-aza- (lactam) steroidal alkylating esters exert their cytostatic and cytotoxic effects on cancer cells combining multiple pharmacological functions, including the induction of genotoxic effects and DNA damage through their alkylating moiety, as well as the inhibition of PARP activity by binding on its catalytic site [17,18,19,20,21].

## 2. Materials and Methods

### 2.1. Compounds

**Figure 1:**
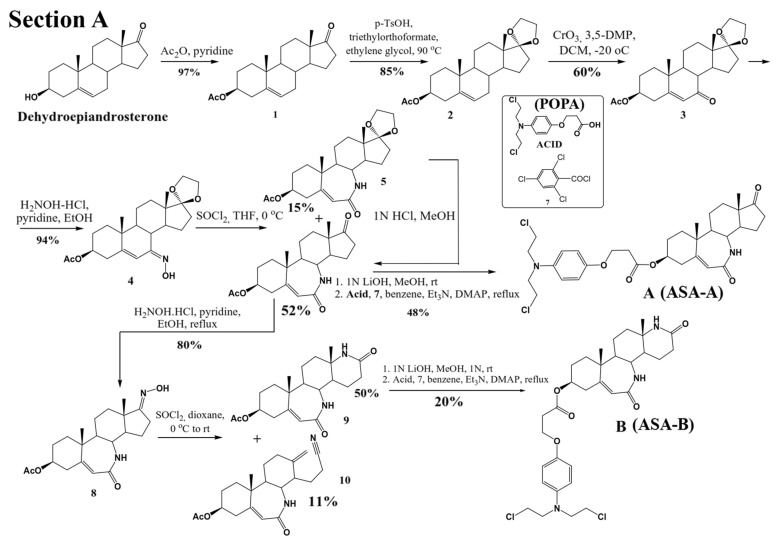

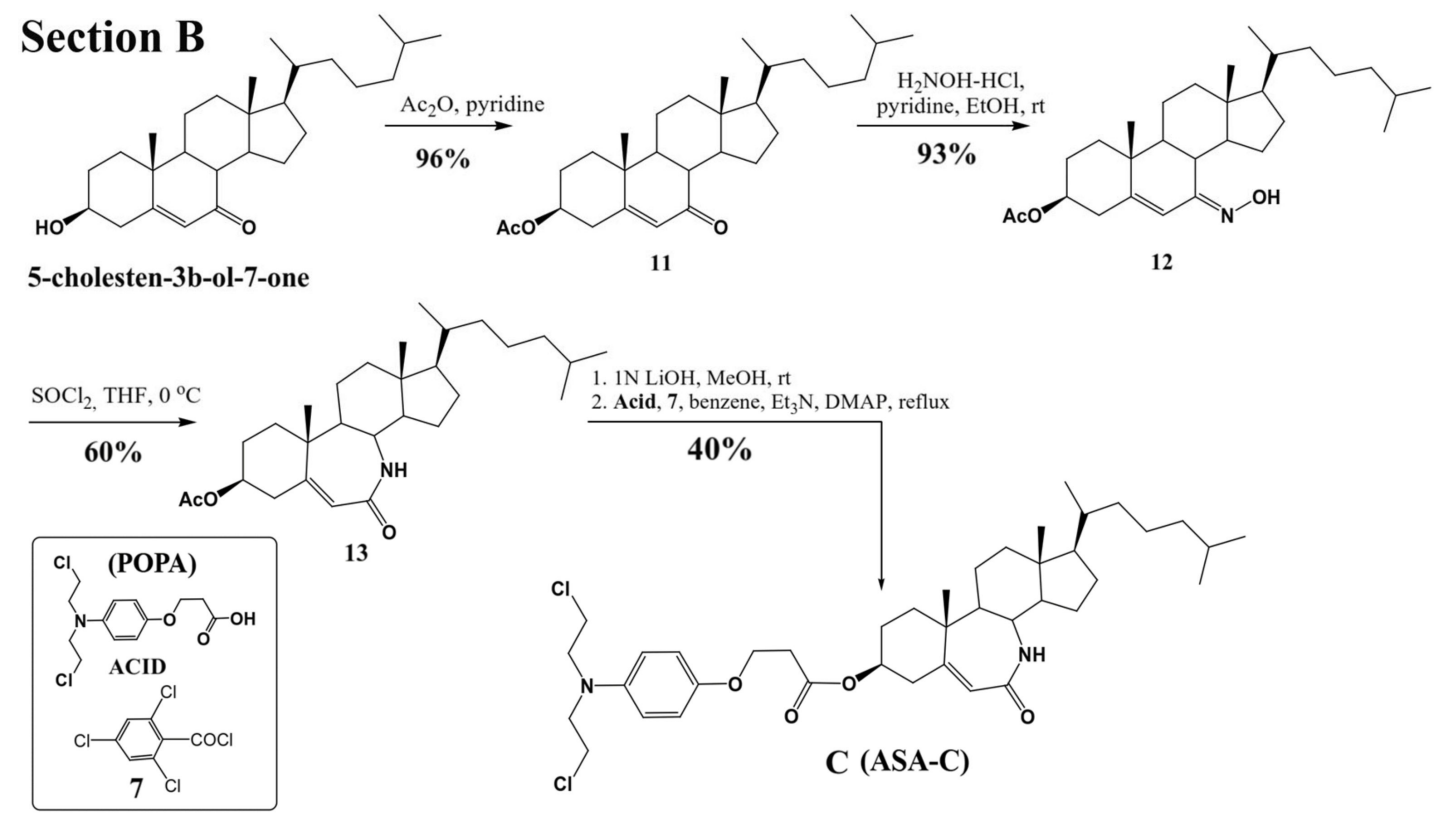
Schematic outlining the synthetic procedure and respective chemical reaction yields of B-homo-aza (lactam)-alkylator (ASA-A), B,D-homo-aza (bilactam)-steroid alkylator (ASA-B) derived from dehydroepiandrosterone (**Section A**) and B-homo-aza (lactam)-alkylator (ASA-C) derived from 5-cholesten-3b-ol-7-one (**Section B**), esterified with the alkylating acid 3-{4-[bis (2-chloroethyl) amino] phenoxy}propanoic acid (POPA).

Three lactamic homo-aza-steroidal alkylators—the B-lactam-steroid alkylator (derived from dehydroepiandrosterone), namely 7a,9a-dimethyl-2,10-dioxo-1,2,4,5,6,7,7a,7b, 8,9,9a,10,11,12,12a,12b-hexadecahydrobenzo[d]indeno [4,5-b]azepin-5-yl 3-(4-(bis(2-chloroethyl)amino)phenoxy)propanoate (ASA-A), the B,D-homo-aza (bilactam)-steroid alkylator (derived from dehydroepiandrosterone), namely 11a,13a-dimethyl-2,6-dioxo-2,3,4,4a,4b,5,6,8,9,10,11,11a,11b,12,13,13a-hexadecahydro-1H-benzo[4,5]azepino [2,3-f]quinolin-9-yl 3-(4-(bis(2-chloroethyl)amino)phenoxy)propanoate (ASA-B), and the B-homo-aza (lactam)-alkylator derived from 5-cholesten-3b-ol-7-one (ASA-C), namely 7a,9a-dimethyl-10-((R)-6-methylheptan-2-yl)-2-oxo-1,2,4,5,6,7,7a,7b,8,9,9a,10,11,12,12a,12b-hexadecahydrobenzo[d]indeno[4,5-b]azepin-5-yl 3-(4-(bis(2-chloro ethyl)amino)phenoxy)propanoate were synthesized and prepared according to previously described methods and procedures (Figure 1) [17,22,23,24]. All three alkylating B-lactam steroidal esters were esterified with the alkylating nitrogen mustard 3-(4-(bis(2-chloroethyl)amino)phenoxy)propanoic acid (POPA).

Furthermore, the third-generation well-established PARP inhibitor Olaparib (AZD2281, AstraZeneca Pharmaceuticals) was tested and used as a positive control.

Stock solutions of the tested compounds were made immediately before use. The compounds were initially dissolved in a small volume of 10% dimethyl sulfoxide (DMSO). Afterwards, they were diluted in a culture medium to reach the final concentrations of 0.05–100 μM for in vitro testing. The solvent (DMSO) in the highest concentration used in the test did not reveal any cytotoxic activity.

### 2.2. Cell Lines

All human ovarian cancer cell lines described below and used for this study were obtained from the American Type Culture Collection (ATCC^®^).

UWB1.289 (CRL-2945™) and UWB1.289+BRCA1 (CRL 2946™) cell lines were cultured in 50% RPMI 1640 and 50% MEGM medium supplemented with 1% antibiotics (penicillin/streptomycin) and 6% fetal bovine serum. The mediums used for OVCAR-3 and SKOV-3 cells are RPMI 1640 and McCoy’s 5A Medium, respectively, containing 10% *v/v* of fetal bovine serum (Gibco FBS, Thermo Fisher Scientific Inc., Waltham, MA, USA,) and a mixture of antibiotics (100 IU/mL penicillin and 100 μg/mL streptomycin). Moreover, the normal human lung fibroblasts MRC-5 cells (CCL-171™) were cultured in RPMI 1640 medium and tested in order to prove the compound selectivity versus the ovarian cancer cells. All mediums used were sterilized by filtration (Corning-Costar filter, diameter 0.2 μm). The cell cultures were maintained at 37 °C in a humid atmosphere saturated with 5% CO_2_.

The in vitro experiments included the analysis of the compounds’ biological activity and efficacy on four studied human ovarian cancer cell lines; these cell lines were selected based on their characteristics, such as their genetic background and chemotherapeutic resistance (Table 1). These selection criteria are considered essential in order to facilitate the investigation of ovarian cancer drug resistance and the evaluation of potential therapeutic protocols.

Both UWB1.289 and UWB1.289 + BRCA1 carry mutations in the tumor suppressor gene TP53 similar to the BRCA1-null breast cancer cell line HCC1937. UWB1.289 and UWB1.289 + BRCA1 have a negative expression of progesterone, estrogen and androgen receptors. UWB1.289 carries a germline BRCA1 mutation within exon 11 and has a deletion of the wild-type allele. In the UWB1.289 + BRCA1 cell line, wild-type BRCA1 is restored [25,26].

### 2.3. MTT Assay

The cells were plated in 96-well plates at a density of 1 × 10^4^ cells/mL per well. They were grown as monolayers for 24 h before treatment with 0.1–100 µM of the compounds ASA-A, ASA-B, ASA-C, POPA, 3-AB and Olaparib for 48 h. The whole duration of the procedure was 72 h, during which the cells were maintained in an incubator. The viability of the cultured cells was estimated by (3-(4,5-dimethylthiazol-2-yl)-2,5-diphenyltetrazolium bromide (MTT) metabolic assay as described previously [27,28,29,30]. MTT (Sigma, St Louis, MO, USA) was dissolved in PBS at a concentration of 5 mg/mL, filter sterilized, and stored at 4oC. MTT (0.2 mL of stock solution) was added to each culture (per mL) and incubated for 3 h at 37 °C to allow metabolism. Formazan crystals were solubilized by acidic isopropanol (0.04 N HCl in absolute isopropanol in a ratio 1:3 *v/v*). The absorbance of the converted dye was measured at a 540 nm wavelength on an ELISA reader (Versamax, Orleans, MA, USA). The mean concentrations of each drug that generated 50% or total (100%) growth inhibition (GI50 and TGI, respectively) and the drug concentrations that produced cytotoxicity against 50% of the cultured cells [(half maximal inhibitory concentration (IC50)] were calculated using the linear regression method [27]. Using seven absorbance measurements [time 24 h(Ct24), control growth 72 h(Ct72) and test growth in the presence of the drug at 5 concentration levels (Tt72x)], the percentage of growth was calculated at each level of the drug concentrations. The percentage growth inhibition was calculated according to the National Cancer Institute (NCI) as: [(Tt72x) − (Ct24)/(Ct72) − (Ct24)] × 100 for concentrations for which Tt72x > Ct24 and [(Tt72x)-(Ct24)/Ct24] × 100 for concentrations for which Tt72x < Ct24. GI50 was calculated from [(Tt72x) − (Ct24)/(Ct72)-(Ct24)] × 100 = 50, TGI from [(Tt72x) − (Ct24)/(Ct72) − (Ct24)] × 100 = 0, and IC50 from [(Tt72x) − (Ct24)/Ct24] × 100 = 50. All the experiments were carried out in triplicate.

### 2.4. PARP-1/2 Activity Assay

**Figure 2:**
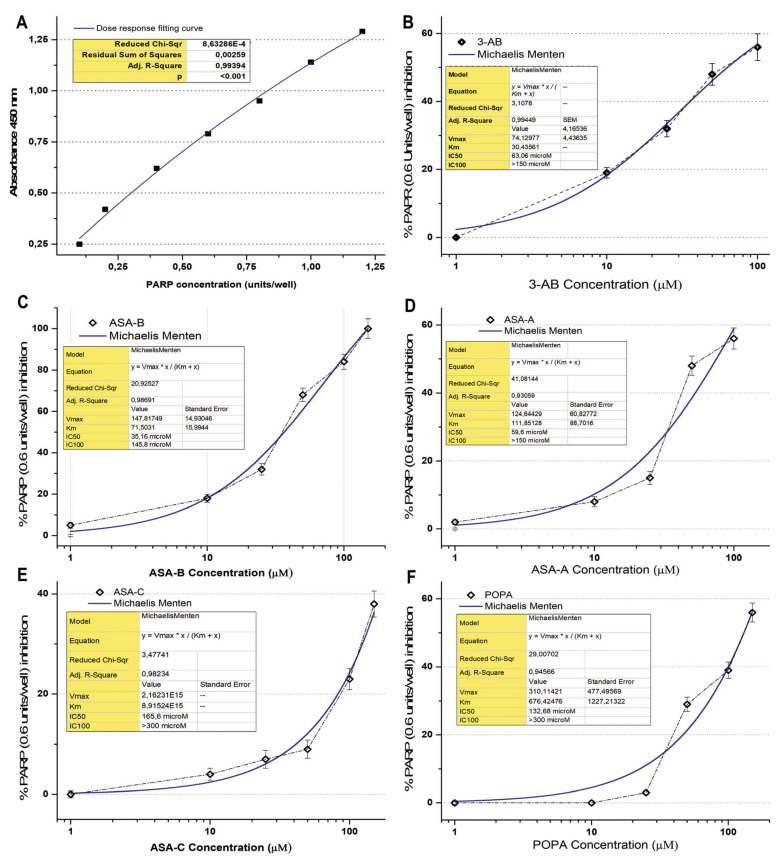
(**A**) Graphical representation of the colorimetric readout of the PARP dose response standard curve (Adj. R-square 0.994, *p* < 0.001); (**B**) PARP (0.6 units/well) inhibition curve and respective kinetics data analysis according to Michaelis Menten’s model for 3-aminobenzamide (3-AB); (**C**) PARP (0.6 units/well) inhibition curve and respective kinetics data analysis according to Michaelis Menten’s model induced by the B,D-bilactam, dehydroepiandrosterone-derived, steroid alkylator ASA-B; (**D**) PARP (0.6 units/well) inhibition curve and respective kinetics data analysis according to Michaelis Menten’s model induced by the B-lactam, dehydroepiandrosterone-derived, steroid alkylator ASA-A; (**E**) PARP (0.6 units/well) inhibition curve and respective kinetics data analysis according to Michaelis Menten’s model induced by the B-lactam, cholesten-derived, steroid alkylator ASA-C; (**F**) PARP (0.6 units/well) inhibition curve and respective kinetics’ data analysis according to Michaelis Menten’s model induced by the conjugant alkylator POPA.

The Universal PARP Colorimetric Assay Kit (Trevigen^®^ Inc., Gaithersburg, MD, USA) was used in order to evaluate the inhibition effect of the tested chemical compounds on PARP1/2 activity. PARP enzymatic activity was assayed according to the manufacturer’s instructions and as previously described [31,32]. The method is based on the incorporation of biotinylated poly (ADP-ribose) (PAR) onto histone proteins in a 96-stripwell microplate format. Colorimetric readouts at 450 nm absorbance of the PARP activity in a serial of PARP dilutions (units/well) were performed, and a standard curve was prepared (Figure 2). 3-Aminobenzamide (3-AB), a well-known inhibitor of PARP enzymes, was used comparatively as a positive control for testing the inhibitory activity on PARP. The compounds ASA-A, ASA-B, ASA-C and POPA were tested in comparison to the standard well-established PARP inhibitor 3-AB at concentrations of 1–300 μM, for the inhibition of PARP activity (0.6 Unit/well PARP-HSA Enzyme). The tested compounds were loaded in quadruplicate into a 96-well plate coated with histones and biotinylated poly ADP-ribose and allowed to incubate for 1 h. Then, they were treated with horseradish peroxidase (HRP) conjugated streptavidin (strep-HRP) and read in a spectrophotometer at 450 nm [25,33].

### 2.5. PARP1 and PARP2 mRNA Expression Analysis

RNA isolation: Cells growing exponentially in culture were treated with 25 μΜ and 50 μΜ of each of the tested compounds for 24 h and then washed in phosphate buffered saline (PBS). The total RNA was extracted using the RNA extraction kit, according to the standard protocols. RNA integrity was assessed by denaturing formaldehyde 1.5% agarose gel electrophoresis and quantified by a spectrophotometer at A260/280 nm.

For each sample in duplicate, 1 μg of checked quality RNA was used to synthetize cDNA with M-MLV reverse transcriptase (RT) (Invitrogen, Thermo Fisher Scientific Inc., Waltham, MA, USA) and 100 pmol/μL hexamer random primers (Invitrogen). An RT reaction mix was incubated for 50 min at 37 °C. The cDNA yield was assessed after 1:10 dilution, by a regular PCR using β-actin (β-ACT) primers. No genomic contamination was detected.

Primers for the genes of interest, PARP1 and PARP2, were designed using Primer3 software (http://biotools.umassmed.edu/bioapps/primer3_www.cgi, accessed on 10 May 2021), based on the complete cDNA sequences deposited in GenBank. Both primers to each set were into different exons to avoid the amplification of contaminating genomic DNA and to eliminate mis-priming events generating a detectable signal. The specificity of the primers and the singularity of the amplified region were verified using http://www.ncbi.nlm.nih.gov/BLAST/, accessed on 10 May 2021 and http://genome.ucsc.edu/cgi-bin/hgBlat?command = start respectively, accessed on 10 May 2021. The amplified fragments were planned to correspond to common regions among different isoforms for each gene [34]. The sequences of primers as designed for real-time PCR were: β-actin (ACTB)/224 bp (BC014861): F 5′AGG ATG CAG AAG GAG ATC ACT G 3′/R 5′GGG TGT AAC GCA ACT AAG TCA TAG 3′; PARP1/94 bp (NM 001618.3): PARP1_F 5′CCTGATCCCCCACGACTTT 3′/PARP1_R 5′GCAGGTTGTC AAGCATTTC 3′; PARP2/229 bp (NM 005484.3): PARP2_10F-CTCCTCC ACTAATCCGGACA/PARP2_12 R-GTGTGGGAGCATGGGTAGAT.

The qRT-PCR was carried out in triplicate for each individual gene of each sample, and all reactions were performed twice by means of the SYBR-Green fluorescent dye detection system (Molecular Probes, Eugene, OR, USA) on a thermocycler- BIORAD CFX96. The reaction volume was 20 μL: 1 μL CDNA, 0.4 μL Forward primer (5 pmol/μL), 0.4 μL Reverse primer (5 pmol/μL), 10 μL buffer 2x (QIAGEN). Forty cycles of PCR amplification were run with 95 °C for 15 min of enzyme activation, 95 °C for 5 s for denaturation, at 59, and 15 s for annealing. The PCR products were directly monitored by measuring the increase of fluorescence due to the binding of the SYBR Green to double-stranded DNA. Melting curve experiments had previously established that the fluorescence signal for each amplicon was derived from the products only, and no primer dimers were found.

The relative gene expression between the treated and untreated samples was calculated using the 2-ΔCT method. The fractional cycle number (CT) expresses the fluorescent signal reached above the detection threshold (minimum detection level). The ΔCT was computed by calculating the difference of the average CT between target genes and the reference β-ACT (ACTB). Due to the exponential nature of PCR, the ΔCT is converted to a linear form by a 2-ΔCT or fourfold difference. RNA samples from treated cell lines versus untreated samples were analyzed. The values of the untreated cells’ mRNA expression were set as 1 for all studied genes [17,34,35].

The absolute gene expression among untreated cell lines was carried out by calculating the ratio of the number of transcripts of the target gene to that of the reference gene, ACTB. This comparison provides the exact number of the PARP1 and PARP2 transcripts in each cell line. The real-time PCR efficiencies were calculated from the slope. The amplification efficiency was similar (1.984–1.986) between the targets and the reference. Finally, all cDNA samples were made from the same amount of RNA (1 μg) and diluted to the same volume.

The PARP1, PARP2 and ACTB absolute mRNA cellular content as well as the relative PARP1/ACTB and PARP2/ACTB mRNA cellular content (± Standard Error, SE) were assessed, as they were induced by the treatment of UWB1.289, UWB1.289 + BRCA1, SKOV-3 and OVCAR-3 human ovarian cancer cells with ASA-A, ASA-B, ASA-C and POPA at 20 and 40 μΜ concentrations, respectively, for 72 h and compared to the corresponding control values (untreated cells).

The data are presented as mean value (MV) ± SE. A statistical analysis was conducted with a one-paired *t*-student test (*p* < 0.01). Group differences were analyzed using the standard Student *t*-test. *p* < 0.01 was considered statistically significant. All experiments were performed in triplicate.

### 2.6. Genotoxicity Assessment–Sister Chromatid Exchanges (SCEs) Assay

Heparinized peripheral blood samples from 5 healthy donors were obtained. Peripheral blood mononuclear cells (PBMCs) were isolated using density gradient centrifugation through Ficoll-Hypaque. The PBMCs were cultured at a density of 1.5 × 10^5^ cells/mL in McCoy’s 5A medium, containing L-glutamine 1% and antibiotics (penicillin 100 U/mL–streptomycin 100 μg/mL), supplemented with 10% heat-inactivated fetal calf serum (FCS) at 37 °C in a humidified 5% CO2 cell culture incubator. T-lymphocytes were stimulated to proliferate with 20 μg/mL phytohaemagglutinin (PHA-L). Moreover, UWB1.289 and UWB1.289 + BRCA1 cells were cultured and maintained as described above. The cultures were set up by adding 1.5 × 10^5^ of cancer cells per mL of the cell culture medium. The PBMC cultures were maintained for 72 h, while UWB1.289 and UWB1.289 were maintained for 144 h. The normal PBMCs and ovarian cancer cells were treated with ASA-A, ASA-B, ASA-C, POPA, 3-AB and olaparib at a 2.5 μΜ drug concentration. The SCEs method was performed as previously described [17,20,21,36]. Briefly, 10 μM of 5- bromodeoxyuridine (5-BrdU) was added to PBMCs cultures at 24 h and to ovarian cancer cell cultures at 48 h for two cell cycles. The cell growth in the presence of 5-BrdU for two rounds of DNA replication was followed by collecting metaphase spreads on glass slides. To prepare the metaphase spreads, exponentially growing cells were treated with 0.2 μg/mL of colcemid for 3–5 h. The cells were collected and incubated in a hypotonic solution (0.56% KCl), fixed in methanol:acetic acid (3:1), spotted to slides and air-dried. To measure chromosomal aberrations, the slides were stained with a 2% Giemsa/Sorensen’s buffer. Differential chromosome staining was achieved by treatment with the UV-sensitive dye Hoechst 33,258 (10 μg/mL), long-wave UV light exposure (for about 1 h and 30 min to long-wave ultra-violet light emitted from a lamp located at a distance of 10 cm from the slides) and Giemsa staining, which gives a permanent record of the exchanges. More than 30 suitably spread second-division cells from each culture were measured on coded slides to establish mean SCE values. The preparations were scored for cells in their first, second and subsequent divisions, with criteria that were previously described, and the cells that were suitably spread were scored blindly for SCEs and cancer cell proliferation kinetics. For the assessment of the Proliferation Rate Index (PRI), at least 100 metaphases were evaluated. The PRI was calculated according to the formula PRI = (M1 + 2M2 + 3M3)/N × 100, where M1, M2 and M3+ indicate the number of metaphases corresponding to the first, second and third or subsequent divisions, respectively, and N is the total number of metaphases scored (M1 + M2 + M3).

## 3. Results

### 3.1. In Vitro Anticancer Activity Testing

The results were expressed as GI50 (μΜ) (drug concentration that induces a 50% inhibition of cell growth) TGI (μΜ) (drug concentration that induces a 100% cell growth inhibition) indicating the cytostatic effects, and IC50 (μΜ) (drug concentration that induces 50% cytocidal effects) indicating the cytotoxic effects of the tested compounds on UWB1.289, UWB1.289 + BRCA1, OVCAR-3 and SKOV-3 human ovarian cancer cells. The tested compounds inhibited the cell growth in a dose-dependent manner. The results are summarized in Table 2. The B- lactam steroid alkylator, ASA-A, and the B,D-bilactam steroid alkylator, ASA-B, were shown to be effective with significantly elevated cytostatic and cytocidal activity, considerably higher (*t*-test, *p* < 0.001) than the aza-steroid alkylator ASA-C and the alkylating component POPA. ASA-C and the alkylating component 3-{4-[bis (2-chloroethyl) amino] phenoxy} propanoic acid (POPA) appeared almost inactive as far as their in vitro cytostatic and cytotoxic effects are concerned, at varying doses (with a maximum of 100 μΜ) on human ovarian cancer cell lines. At the tested drug concentrations, 3-AB showed no significant cytostatic or cytotoxic effects, while olaparib generated cytostatic and no cytocidal effects against the BRCA1 mutated UWB1.289 and a lower cytostatic potential against the OVCAR-3 ovarian cancer cells. In total, the B-lactam steroidal alkylator ASA-A appeared to be more potent than the B,D-bilactam steroid alkylator ASA-B against the human ovarian cancer cells, with the exception of UWB1.289 cells, where ASA-B had a better cytostatic and cytotoxic activity, presenting a synergistic lethality effect. The tested aza-steroidal alkylators ASA-A and ASA-B, in terms of their cytostatic and cytotoxic activity, presented a significant selectivity against the ovarian human cancer cells, since they generated significantly lower cytostatic (IG50 and TGI) and cytotoxic (IC50) effects on normal human lung fibroblasts MRC-5.

The data showed that the compounds under investigation influenced tumor cell growth differentially, demonstrating the BRCA1-null ovarian cancer cell line UWB1.289 to be significantly more sensitive to the antiproliferative effects of the investigated compounds than the respective BRCA1-wild type UWB1.289 + BRCA1 ovarian cancer cell line (*t*-test, *p* < 0.001). The BRCA1 mutated ovarian cell line UWB1.289 appeared to be particularly more sensitive to the cytostatic and cytocidal effects of the tested alkylating agents than the other ovarian cancer cell lines tested in consistence to its BRCAness phenotype (*t*-test, *p* < 0.001). ASA-B showed the greatest anticancer activity against UWB1.289 cells, in vitro (*t*-test, *p* < 0.001), as already mentioned. The second most significant activity against UWB1.289 cancer cells was generated by the B-lactam steroidal alkylator, ASA-A (*t*-test, *p* < 0.001).

The SKOV-3 and OVCAR-3 ovarian cancer cells were shown to be irresponsive to the anticancer activity of alkylators such as Cisplatin and Melphalan, as well as to the chemotherapeutic cytotoxic agent Adriamycin. Accordingly, our experimental data show SKOV-3 and OVCAR-3 cells to be relatively resistant to the anticancer activity of the tested nitrogen mustard alkylating agent POPA. However, this apparent phenotype of the insensitivity of SKOV-3 and OVCAR-3 human ovarian cancer cells is overcome by the lactam steroid alkylators, ASA-A and ASA-B, that produced excellent cytostatic and cytotoxic anticancer effects on these cells (*t*-test, *p* < 0.001).

All four tested cancer cell lines bear different active mutations of the onco-suppressor gene TP53. Both ASA-A and ASA-B appear to procure a remarkable anticancer activity independently of the presence of these mutations on the TP53 gene (*t*-test, *p* < 0.001).

Moreover, SKOV-3 ovarian cancer cells that bear an active mutation on the PIK3CA gene and present a high microsatellite instability (MSI-high), in contrast to the OVCAR-3 and UWB1.289 + BRCA1 ovarian cancer cells that are microsatellite-stable (MSS), demonstrate a clearly higher susceptibility to the anticancer activity of ASA-A and ASA-B as far as their cytostatic effects and the 100% cell growth inhibition (TGI) are concerned (*t*-test, *p* < 0.01).

Finally, the cytostatic or cytotoxic antitumor effects of the homo-aza-steroidal alkylating esters, ASA-A and ASA-B, were observed in both steroid receptors (Estrogen/Progesterone/Androgen receptors), expressing and non-expressing ovarian cancer cell lines (*t*-test, *p* < 0.001).

### 3.2. PARP Enzymatic Activity Inhibition

The B-lactam steroid alkylators ASA-A, ASA-B and ASA-C, as well as the alkylating moiety POPA (nitrogen mustard) were tested for their direct inhibitory effect against the activity of PARP enzymes in a cell-free experimental biochemical assay in comparison to the well-known PARP inhibitor 3-AB. The inhibition curves and the respective kinetics data analysis according to Michaelis Menten’s model for the 3-aminobenzamide (3-AB), B-lactam (dehydroepiandrosterone-derived) steroid alkylator ASA-A, B,D-bilactam (dehydroepiandrosterone-derived) steroid alkylator ASA-B, B-lactam (cholesten-derived) steroid alkylator ASA-C and alkylating component POPA are presented in Figure 2 and Table 3. The B,D-bilactam steroid alkylator ASA-B and the B-lactam steroidal alkylator ASA-A showed significant inhibitory effects on PARP activity (*t*-test, *p* < 0.0001), ASA-B being the most potent inhibitor producing better inhibitory effects on PARP activity than 3-AB (*t*-test, *p* < 0.01). Thus, 3-AB, ASA-A, ASA-B, ASA-C and POPA induce a 50% inhibition of the PARP enzymatic activity (IC50) at concentrations of 63.06, 59.6, 35.16, 165.6 and 132.68 μM, respectively (Table 3). ASA-B and ASA-A exert their inhibitory effects on PARP activity, generating much higher kinetic parameters of Km and Vmax than 3-AB (*t*-test, *p* < 0.01). Contrarily, ASA-C appeared as a noticeably weak inhibitor of PARP activity, with high IC50 and very low Km and Vmax. Moreover, POPA acts like an inhibitor of PARP activity at significantly higher concentrations, with a relatively high IC50 and the highest Km and Vmax inhibition kinetic parameters. On the other hand, Olaparib generated by far the most potent inhibitory effect on PARP activity, demonstrating an IC50 at 0.95 μΜ and an IC100 at 13.17 μΜ, respectively (Table 3).

**Figure 3:**
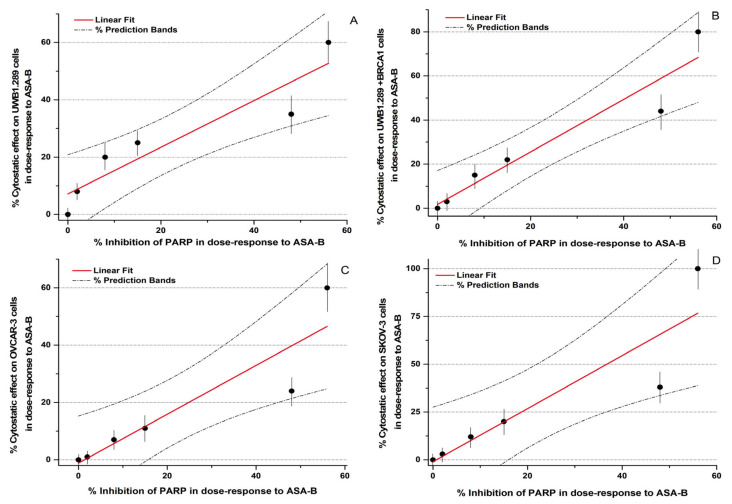
Correlation curves and 95% prediction bands between %PARP inhibition and %cytostatic effects in dose-response to the B,D-bilactam steroid alkylator ASA-B on UWB1.289 (**A**), UWB1.289+ BRCA1 (**B**), OVCAR-3 (**C**) and SKOV-3 (**D**) human ovarian cancer cells.

In conclusion, the potency of inhibition of the PARP activity induced by ASA-A and ASA-B is notably correlated to their cytostatic effects on UWB1.289, UWB1.289 + BRCA1, OVCAR-3 and SKOV-3 human ovarian cancer cells (Figure 3, Table 4). The respective Pearson’s correlation indexes (r) between cytostatic activity and PARP inhibitory effects for ASA-A and ASA-B are 0.95 and 0.93, respectively, at a significance level *p* < 0.01. On the other hand, the corresponding correlations for ASA-C and POPA are low and not significant (NS) (Figure 3, Table 4), while the 3-AB induced cytostatic effects were significantly correlated with PARP inhibition only on BRCA1 mutated (presenting “BRCAness”) UWB1.289 human ovarian cancer cells (Table 4).

### 3.3. PARP1 and PARP2 Expression

**Figure 4:**
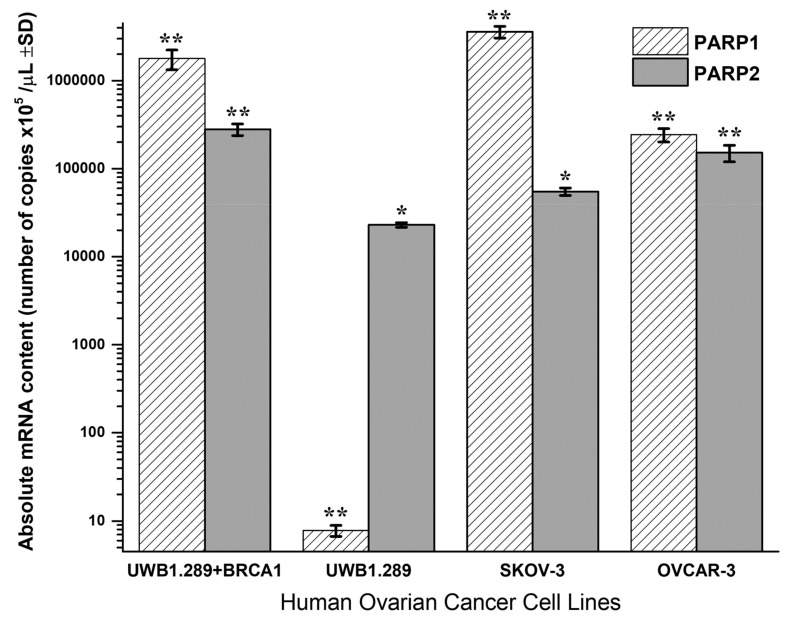
Absolute mRNA cellular content and transcription of PARP1 and PARP2 (± SD) in untreated UWB1.289 + BRCA1, UWB1.289, SKOV-3 and OVCAR-3 human ovarian cancer cell cultures at the exponential phase of cell growth. Significance levels in between PARP1 or PARP2 mRNA transcription in the tested ovarian cancer cell lines were denoted, (**) for *p* < 0.0001 and (*) for *p*< 0.001.

Quantitative PCR analysis of PARP1 and PARP2 absolute mRNA content in UWB1.289 + BRCA1, UWB1.289, OVCAR-3 and SKOV-3 untreated human ovarian cancer cell lines is presented in Figure 4.

BRCA1-null UWB1.289 cancer cells transcribe a significantly higher PARP2 (*t*-test, *p* < 0.001) and a strikingly lower PARP1 mRNA content (*t*-test, *p* < 0.001) due to the BRCA1 loss of activity. Contrariwise, the wild-type UWB1.289 + BRCA1 cells presented normally a high PARP1 and a lower PARP2 expression (*t*-test, *p* < 0.001). In general, the UWB1.289 + BRCA1, OVCAR-3 and SKOV-3 cells showed a natural and significantly higher PARP1 and a lower PARP2 expression. Comparatively, the BRCA1 mutated UWB1.289 cancer cells demonstrated a much lower PARP1 and a reduced PARP2 mRNA transcription than the other ovarian cancer cell lines tested (*t*-test, *p* < 0.001).

**Figure 5:**
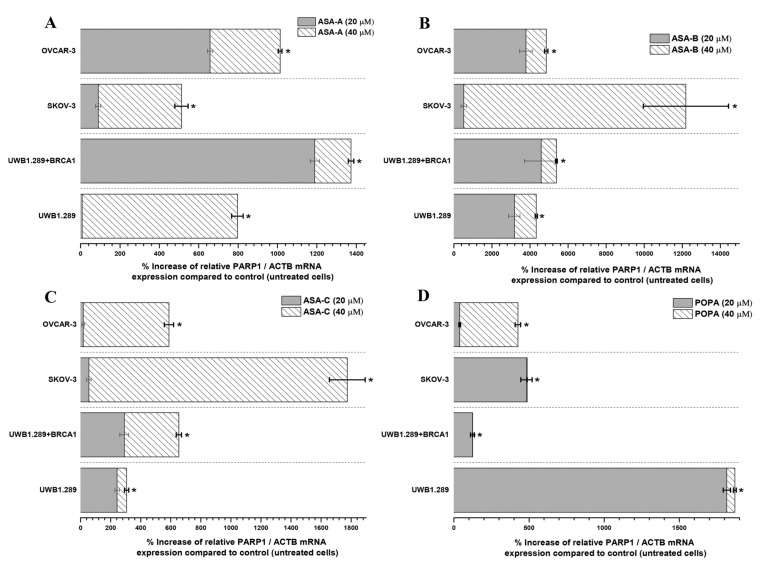
% Increase of the relative PARP1/ACTB mRNA cellular content (± Standard Error, SE) as compared to the corresponding control values (untreated cells) induced by the treatment of UWB1.289, UWB1.289 + BRCA1, SKOV-3 and OVCAR-3 human ovarian cancer cells with ASA-A (**A**), ASA-B (**B**), ASA-C (**C**) and POPA (**D**) at 20 and 40 μΜ concentrations, respectively, for 72 h. (*) Statistical significance for *p* < 0.001 (two-tail paired *t*-test).

**Figure 6:**
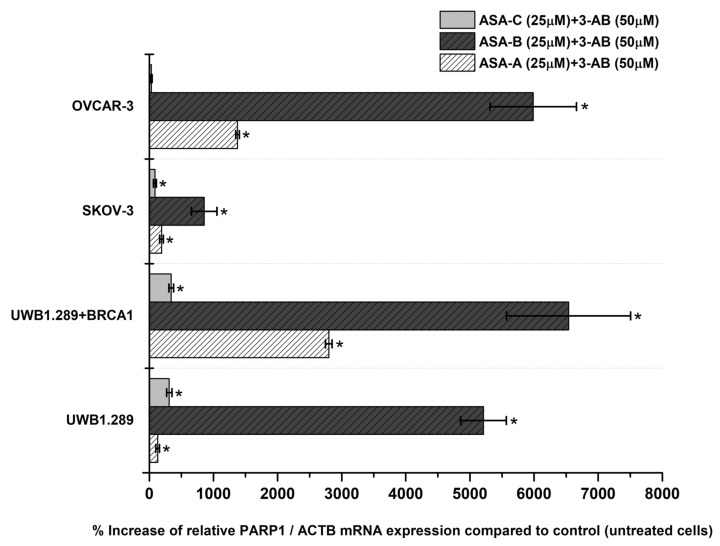
% Increase of the relative PARP1/ACTB mRNA cellular content (± Standard Error, SE) as compared to the corresponding control values (untreated cells) induced by the treatment of UWB1.289, UWB1.289 + BRCA1, SKOV-3 and OVCAR-3 human ovarian cancer cells with the combination of ASA-A, ASA-B or ASA-C at 25 μM concentration along with 3-AB at 50 μM concentration for 72 h. (*) Statistical significance for *p* < 0.001 (two-tail paired *t*-test).

**Figure 7:**
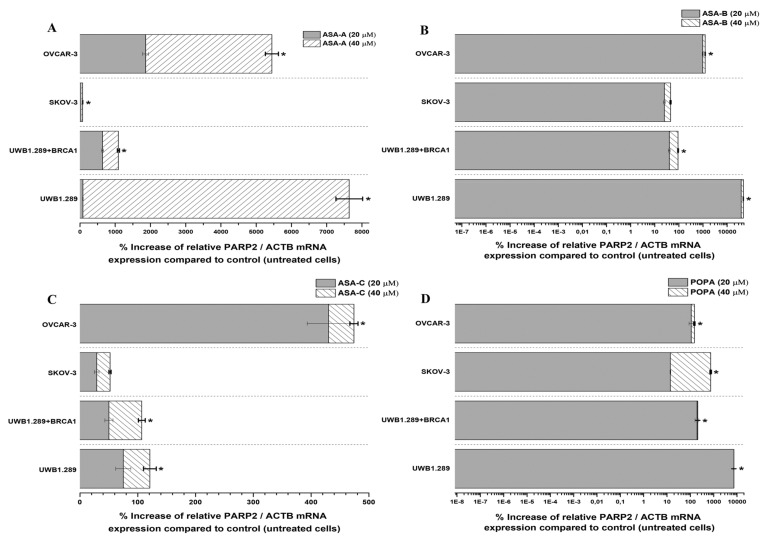
% Increase of the relative PARP2/ACTB mRNA cellular content (± Standard Error, SE) as compared to the corresponding control values (untreated cells) induced by the treatment of UWB1.289, UWB1.289 + BRCA1, SKOV-3 and OVCAR-3 human ovarian cancer cells with ASA-A (**A**), ASA-B (**B**), ASA-C (**C**) and POPA (**D**) at 20 and 40 μΜ concentrations, respectively, for 72 h. (*) Statistical significance for *p* < 0.001 (two-tail paired *t*-test).

The changes (%) in the relative PARP1/ACTB and PARP2/ACTB mRNA cellular content (± Standard Error, SE) as compared to the corresponding control values (untreated cells) induced by the treatment of UWB1.289, UWB1.289 + BRCA1, SKOV-3 and OVCAR-3 human ovarian cancer cells with ASA-A, ASA-B, ASA-C and POPA at 20 and 40 μΜ concentrations for 72 h, as demonstrated by the quantitative PCR analysis, are presented in Figure 5, Figure 7, respectively. Moreover, a similar expression analysis is described in Figure 6, regarding the treatment of the same tested human ovarian cancer lines with a 3-AB agent in combination with ASA-A, ASA-B or ASA-C, enclosing the respective alterations on the relative PARP1/ACTB mRNA cellular content.

In general, the results indicated that the PARP1/ACTB and PARP2/ACTB mRNA cellular content ratios are notably increased (*t*-test, *p* < 0.001) in most cases and for all tested cancer cell lines after treatment with the alkylators ASA-A, ASA-B, ASA-C and POPA. However, these rises were obviously differentiated depending on the variant cell types and biology, as well as due to the differential cell sensitivity in the tested compounds. Consequently, as was revealed, the more sensitive a cancer cell line to the cytostatic and cytotoxic effects of a tested compound, the higher the increase of the relative PARP1/ACTB and PARP2/ACTB mRNA ratios observed.

The highest increases of PARP1/ACTB mRNA ratios were induced by the most potent PARP inhibitor, the bilactamic steroid alkylator ASA-B, reaching at 120-fold increase in SKOV-3 and a 42–50-fold increase in UWB1.289, UWB1.289 + BRCA1 and OVCAR-3 human ovarian cancer cells. ASA-A appeared as the second most potent compound to produce a PARP1/ACTB mRNA ratio increment, while the POPA and ASE-C were the least potent, respectively. The aza-steroidal alkylators, ASA-A, ASA-B and ASA-C induced a conspicuously higher increase of the PARP1/ACTB mRNA ratio in the UWB1.289 + BRCA1 than in the BRCA1 mutated UWB1.289 cancer cells. On the other hand, POPA was the best augmenter of the PARP1/ACTB mRNA ratio in BRCA1-null UWB1.289 cancer cells (Figure 5).

The simultaneous treatment of the UWB1.289, UWB1.289 + BRCA1, SKOV-3 and OVCAR-3 human ovarian cancer cells with 3-AB in combination with ASA-A, ASA-B or ASA-C induced a significant (*p* < 0.001) synergistic effect on the % increase of PARP1/ACTB mRNA cellular content, mostly in ASA-B and less in ASA-A combinations with 3-AB. The treatment with combinations of 3-AB with ASA-C produced obviously lower increases, as well as synergistic effects (Figure 6). The treatment of the tested cancer cell lines with the 3-AB agent alone generated non-significant alterations in the PARP1/ACTB mRNA cellular content (data not shown).

As far as the inducible increases of PARP2/ACTB mRNA ratios are concerned, ASA-A, ASA-B and POPA were comparably more effective, whereas ASA-C was less effective against all tested cancer cell lines (Figure 7). ASA-A, ASA-B and POPA brought about a considerably higher increase of the PARP2/ACTB mRNA ratio in the BRCA1-null UWB1.289 than in the BRCA1 wild-type UWB1.289 + BRCA1 cancer cells, with the ASA-B begetting a 140- to150-fold increase of the PARP2/ACTB mRNA ratio in UWB1.289 cells.

ASA-A, ASA-B, ASA-C and POPA generated a statistically significant enhancement and increase of the PARP1 and PARP2 mRNA expression in all tested cancer cell lines. These material results on the regulation of PARP1/2 transcription were differential and depended on the drug’s PARP inhibitory activity, the drug concentration, the cell type and the cancer cells’ sensitivity for each tested compound.

All tested chemical derivatives produced a similar outcome onto UWB1.289 + BRCA1, UWB1.289, SKOV-3 and OVCAR-3 human ovarian cancer cells, with the activity of the lactam steroid alkylators ASA-A and ASA-B being more profound than the alkylating agent POPA. Furthermore, it is notable that the tested compounds induced a higher increase of the expression of PARP1 in the BRCA1 wild-type UWB1.289 + BRCA1 than in BRCA1-null UWB1.289 cells, whilst the higher increase of the expression of PARP2 was observed in UWB1.289 cells.

### 3.4. Genotoxicity Assessment–Sister Chromatid Exchanges (SCEs)

The B-lactam- steroid alkylators ASA-A, ASA-B and ASA-C, as well as the alkylating moiety POPA and the PARP inhibitors 3-AB and Olaparib were tested for their genotoxic effects on PHA-stimulated T-lymphocytes obtained from five healthy donors, as well as on UWB1.289 and UWB1.289 +BRCA1 human ovarian cancer cells at a concentration of 3 μM.

ASA-A and ASA-B induced a significant (*p* < 0.01) increase of SCEs and a significant decrease of PRIs in all the cases tested. ASA-B was the most potent genotoxic agent. On the other hand, ASA-C and POPA showed no significant genotoxic or cytostatic effects at the tested drug concentration. The PARP inhibitor 3-AB induced a significant increase of SCEs in all cases but no cytostatic effects were observed. The well-established potent PARP inhibitor, Olaparib, exhibited a significantly high genotoxic activity in all cases, and in particular against the BRCA1 mutated UWB1.289 ovarian cancer cells. Moreover, Olaparib induced the attenuation of PRI and cytostatic effects only against UWB1.289 cells.

BRCA1-defective UWB1.289 cells showed a certain sensitivity to the active tested compounds, and in particular to B-lactam steroid alkylators ASA-B and ASA-A, and a significant but lower sensitivity to Olaparib at the tested drug concentration. ASA-B performed with the highest activity against the UWB1.289 cells. The results are shown in Table 5.

## 4. Discussion

PARP-1, a multifunctional nucleus protein with a multidomain structure, has well documented roles in DNA damage repair, necrosis, apoptosis and DNA damage-dependent cancer progression. Recent studies also associate it with epithelial to mesenchymal transition (EMT), a biological phenomenon involved in the early progression of cancer metastasis, and the modulation of EMT regulators like Vimentin, Claudin-1 and other transcription factors, playing a dual role in EMT. In this sense, PARP-1 became a special target for cancer treatment, and several inhibitors of PARP-1 like Olaparib, Rucaparib and Veliparib are already in use [8].

Ovarian cancer is a disease with an extreme genetic complexity and a defective DNA repair via a homologous recombination (HR) pathway. Apart from BRCA1/2 mutations, additional mechanisms can also result in HR pathway alterations and, consequently, lead to a clinical benefit from PARP inhibitors. Thus, inhibitors of PARP have a significant therapeutic impact on the treatment of women with epithelial ovarian cancers, and in particular those with the most common histological subtype, high-grade serous cancer, because of the high rate of homologous recombination (HR) deficiency. The inhibition of PARP generates anticancer effects by disrupting DNA repair, thus causing genomic decay. PARP inhibitors are approved as a frontline maintenance treatment for patients with and without BRCA-associated cancers, and clinical data demonstrate the effectiveness of PARP inhibition in women with recurrent epithelial ovarian cancer harboring BRCA1/2 mutations and those with platinum-sensitive disease as a maintenance therapy or as a second-line treatment in recurrence [37,38].

It has currently been shown that the hybrid aza- (lactam) steroidal alkylating esters, which are chimeric steroid compounds, generate a dual inhibition on the RAS/PI3K/AKT and RAS/RAF/MEK/ERK signaling pathways [23]. In our case, the studied lactam steroidal alkylators ASA-A and ASA-B produced a significantly high anticancer activity in vitro, over against ASA-C, that was almost inactive. As previously reported [28,30,39,40] and demonstrated by our data, the existence of the hydrocarbon side chain on the C-17 position constrains the anticancer pharmacological effects and nearly inactivates the steroidal alkylating esters, probably due to the occurrence of some biochemical obstruction. In addition, the PARP-inhibitor-induced activation of the cytoprotective PI3K-Akt pathway likely contributes to the development of PARP inhibitor resistance. Recently, the molecular mechanism that explains how PARP inhibition induces Akt activation and may account for apoptosis resistance and mitochondrial protection in oxidative stress and in cancer has been discovered [41]. However, as previously reported [23], A- and D-lactam steroid alkylators (similar to the B-lactam-steroid alkylating agents tested in the present report) generate, apart from the suspension of PARP activation, a dual inhibition on the RAS/PI3K/AKT and RAS/RAF/MEK/ERK signal pathways, abrogating the undesirable PARP-inhibition-induced activation of the PI3K-Akt pathway, deploying a unique pharmacological molecular action. Furthermore, the inhibitory effects of the homo-aza-steroidal alkylating esters on the RAS/RAF/MEK/ERK signaling pathway induce a further indirect interruption of PARP-1 activation. As previously reported [42], PARP-1 is activated via a direct interaction with phosphorylated ERK2, and ERK2-induced PARP-1 activation dramatically amplifies ERK-signals, enhancing ERK-induced phosphorylation. Hence, PARP-1 activation mediates epigenetic mechanisms promoting growth, proliferation and differentiation regulated by the Raf-MEK-ERK phosphorylation cascade.

The B-lactam ASA-A and B,D-bilactam ASA-B steroid alkylators, unlike the nitrogen mustard alkylator POPA, presented excellent cytostatic and cytocidal activity against SKOV-3 and OVCAR-3 human ovarian cancer cells, which are insensitive to the anticancer activity of alkylators such as cisplatin and melphalan, as well as to the chemotherapeutic cytotoxic agent Adriamycin. Moreover, these active compounds show a selectivity of their cytostatic and cytotoxic effects against the ovarian cancer cells. These results further confirm that the hybrid aza-steroid alkylators are overcoming relevant cell drug resistance pharmacological mechanisms. Moreover, these chimeric compounds, that bear a modified steroid containing a lactamic group (-NH-C=O−) and carry an alkylating moiety, produce a higher antitumor activity than other alkylators conventionally used in cancer therapy [16,22,24,39,40,43,44].

Evidently, comparing the cell lines tested with the respective compounds, the anticancer outcome of ASA-A and ASA-B proved independent of the TP53 mutations borne by cancer cells. Moreover, cancer cells that have an active mutation on the PIK3CA gene and/or present a high microsatellite instability (MSI-high) seem to demonstrate a higher sensitivity to active lactam steroidal alkylators [39,40]. These are noteworthy observations because TP53 mutations are instrumental in the development of cancer cell resistance to chemotherapeutic drugs, such as classical cytotoxic agents including cisplatin, doxorubicin, 5-fluorouracil, temozolomide and paclitaxel, since the p53-based chemoresistance is highly associated with the chemical properties of the anticancer drug, the cellular drug target, the biological function being blocked by the chemotherapeutic agent, the genomic instability and alterations of the tumor, as well as its differentiation state [45]. It has been reported that the inactivation of TP53 mutations in the presence of BRCA mutations may be associated with cancer cell resistance in the treatment with PARP inhibitors. The synthetic lethality of PARP inhibition in these cases is reported as p53-dependent [45,46,47]. On the other hand, B-lactam-steroidal alkylating agents attain anticancer activity in both p53-mutated and non-mutated cancer cell lines, and this effect may be independent of their PARP inhibitory activity. The broad screening of A-, B- and D-lactam steroid alkylators for anticancer activity against several panels of human cancer cell lines (breast cancer, colon cancer, pancreatic cancer, small-cell-lung cancer cells) showed that these compounds produce their cytostatic and cytotoxic effects independently of the presence of different inactivating mutations of TP53 (data not shown).

It is also interesting to note that mutations of PIK3CA in ovarian and breast cancer have been associated with chemoresistance [48,49] and endocrine resistance to hormonal therapy [50,51]. The sensitivity of PIK3CA mutated cancer cells to the lactam steroidal alkylators is in accordance with previous findings that showed that these hybrid steroid compounds inhibit the PI3K/AKT signaling pathway [23].

As previously described [17,23,37,43] and indicated by our data, the expression of steroid receptors in ovarian cancer cells, contrary to breast cancer, is independent of, and does not affect, the cytostatic or cytotoxic antitumor effect of the homo-aza-steroidal alkylating esters. Earlier findings about the antitumor effects of homo-aza-steroidal alkylators against human breast cancer cells detected a weak to moderate dose-dependent correlation of their anticancer activity with the estrogen receptor expression [37,43].

As previously reported, A- and D-lactam steroidal alkylators are potent inhibitors of PARP activity by binding in PARP’s catalytic site and blocking its activity [17]. Our data showed that the B,D-bilactam ASA-B and the B-lactam ASA-A steroid alkylators also exhibited inhibitory effects on PARP activity in a cell-free assay, with ASA-B prompting even stronger inhibitory effects than the well-established PARP inhibitor 3-AB. These inhibitory effects on PARP activity were significantly correlated to their cytostatic effects on UWB1.289, UWB1.289 + BRCA1, OVCAR-3 and SKOV-3 human ovarian cancer cells. Nevertheless, the well-established PARP inhibitor Olaparib demonstrated much higher inhibitory effects than the tested B-lactam steroid alkylators on PARP activity. However, Olaparib induced cytostatic effects only against the BRCA1-null UWB1.289 and less against OVCAR-3 cancer cells at the tested drug concentrations.

In order to repair the generated genotoxicity and DNA damage induced by the lactam steroidal alkylators ASA-A and ASA-B, the cells of all the examined human ovarian cancer cell lines responded with significantly elevated transcription levels of PARP1 and PARP2 mRNA. These increases of PARP1,2 mRNA were contingent on the diverse cell biology and sensitivity of the tested compounds and were correlated in proportion and respectively with the cytostatic and cytotoxic effects of the investigated agents. Hence, the lactam steroid alkylators ASA-B and ASA-A have equivocal anticancer properties by simultaneously inhibiting PARP activity and increasing the PARP 1 and 2 mRNA expression. On this aspect, it is verified by our experiments that POPA finally obtains a reduced anticancer “state” among the compounds studied; while it provokes alkylating DNA damage and thus an increase in PARP mRNA transcription, it cannot obstruct the specific cell repair mechanism, not being a PARP 1,2 inhibitor. This fact, in combination with its highly toxic nature, leads to its anti-tumor inferiority.

It has been shown as well [21] that the homo-aza-steroidal alkylating esters of nitrogen mustards exerted cytogenetic damage, which was enhanced synergistically in combinational treatment with the PARP inhibitor 3-amino-benzamide (3-AB). It has also been shown that treatment with the homo-aza-steroidal alkylators led to the depletion of cellular NAD, while the addition of 3-AB prevented the drop of NAD levels.

While it is argued that for minor DNA damage, cancer cells activate PARP-1 to help in the cell survival by repairing the mild DNA lesions, for high-level DNA damage PARP-1 is overactivated and uses NAD+ as a donor of ADP ribose for ADP-ribosylation, driving the cell into an imbalance of NAD+/ATP ratio, NAD depletion and eventually resulting in an energy-deprived necrosis [8,52,53]. Remarkably, each cell line seems to be “specialized” on PARP1 and/or PARP2 for the aforementioned repair mechanisms, and a ratio between them proportional to the degree of the DNA damage and the mutagenic effect of each alkylating compound is formed (Figure 5 and Figure 7). This overactivation phenomenon, “emerging” both from PARP1 and PARP2 in all tested cell lines is also emphasized in our results in the wild-type cancer cells (without BRCAness), promoting the NAD depletion/energy cell “starvation” as an alternative procedure leading to cellular death, even for more enduring carcinomas, with a mechanism for these compounds similar to the one reported for lactam-steroidal alkylators [21].

The loss of DNA damage repair pathways is an early and frequent event in tumorigenesis, occurring in 40–50% of many cancer types. The basis of synthetic lethality in cancer therapy are the DNA damage-repair-deficient cancers dependent on backup DNA repair pathways. In cancer, the synthetic lethality mostly relates to pairs of genes, where the inactivation of one and the pharmacological inhibition of the other lead to the death of the cancer cells. The combination of PARP inhibitors and other DNA damage repair inhibitors, or the combination of multiple components of the same pathway, may have great potential for synthetic lethality efficiency. Both genetic and epigenetic alterations may serve as synthetic lethal therapeutic markers. PARP is a critical factor in the repair of single-stranded DNA damage via the base excision repair pathway, and PARP inhibitors have a substantial single-agent antitumor activity by inducing synthetic lethality, especially in tumors harboring deleterious germline or somatic BRCA mutations. PARP inhibition produces single-stranded DNA breaks, which may be repaired by homologous recombination, a process partially dependent on BRCA1 and BRCA2 [54,55,56].

Tumors harboring genetic defects in homologous recombination (HR), a DNA double-strand break repair pathway, are hypersensitive to PARP inhibitors. Unlike HR-defective cells, HR-proficient cells manifest very low cytotoxicity when exposed to PARP inhibitors, although they mount a DNA damage response. PARP1 deficiency is markedly increased when combined with other DNA damage response genetic deficiencies. Sister chromatid exchange (SCE) is a recombination event between identical sister chromatids following DNA replication, typically but not uniformly, and resulting in an equal exchange of genetic information. However, SCEs that occur at offset repeats can lead to the loss or gain of genetic information. Thus, increased SCEs are a measure of hyper-recombination leading to chromatid exchange, whether due to deficiencies in genes involved in the suppression of exchange or to unrepaired DNA damage encountered during DNA replication and the subsequent HR-mediated repair at stalled replication forks. An increase in SCEs correlates with the mutagenic potential and the induced loss of heterozygosity. Olaparib causes marked hypersensitivity in HR-deficient cells and increases SCE frequency in repair-proficient human cells. Less potent PARP inhibitors, which did not demonstrate synthetic lethality in BRCA-deficient tumor cells, have been shown to increase SCEs [57]. The B-lactam steroid alkylators ASA-A and ASA-B, although less potent PARP inhibitors compared to Olaparib, induced a significant increase of SCEs in either repair-proficient or repair-deficient normal and cancerous human cells. ASA-B was presented as the more potent genotoxic agent, including the BRCA1-null ovarian cancer UWB1.289 cells.

In HR-deficient tumors, the “synthetic lethality” strategy for PARP inhibition is dependent on unrepaired endogenous strand breaks that arise during DNA replication and normal cell metabolism. A current therapeutic strategy, investigated in ongoing clinical trials, is the combination of chemotherapeutics that directly induce DNA damage whilst also inhibiting single-strand breaks repair with PARP inhibition [58].

Our data revealed that the active lactam steroid alkylator ASA-B exhibited a significantly higher anticancer activity in the BRCA1-null ovarian cancer cell line UWB1.289 than in the respective BRCA1 wild-type UWB1.289 + BRCA1 ovarian cancer cell line. The BRCA1 mutated ovarian cell line UWB1.289 appeared to be considerably more prone to the cytostatic and cytocidal effects of the tested alkylating agents than the other ovarian cancer cell lines tested due to its BRCAness phenotype. The B,D-dilactam steroidal alkylator ASA-B presented a more clear synthetic lethality attribute in BRCA-1 mutated UWB1.289 ovarian cancer cells due to its corresponding cytostatic and cytotoxic effects in relation to its inhibitory activity on PARP enzymes. On the other hand, the B-lactam steroidal alkylator ASA-A induced the strongest cytostatic and cytotoxic antitumor results in all tested ovarian cancer cell lines, and while it proved a potent PARP inhibitor in a cell free assay, the synthetic lethality in BRCA-1 “null” UWB1.289 cells could not be as evident. Moreover, the B,D-bilactam ASA-B and the B-lactam ASA-A steroid alkylators instigated higher increases of PARP1 mRNA cellular content in the UWB1.289 + BRCA1 than in BRCA1 mutated UWB1.289 cancer cells. In addition, these compounds promoted a more appreciable elevation of the PARP2 mRNA transcription in the BRCA1-null UWB1.289 than BRCA1 wild-type, UWB1.289 + BRCA1 cancer cells.

The silencing of DNA damage repair genes by aberrant epigenetic changes provides new opportunities for synthetic lethal therapy in cancer [59]. These chimeric modified homo-aza-steroidal alkylators produce significant and characteristic anticancer features generated in the cancer cells: on the one hand, DNA damage, and on the other hand, disrupting DNA repair by inhibiting PARP activity and causing important cellular energy pool depletion through a high increase of PARP 1 and 2 mRNA transcription and expression. These hybrid lactam steroid alkylators act with multi-targeted pharmacological mechanisms of PARP “trapping”, combined in one molecule, and can cause enhanced synthetic lethality in cancer cells.

Conclusively, the tested B-lactam steroid alkylators are non-typical inhibitors of PARP and are potentially relevant for clinical use due to their multiple targeting. However, the PARP inhibition by these compounds has only been demonstrated in a cell free assay, and it is currently unclear whether this is relevant or contributes to the effects observed in the cells. Nevertheless, despite their low (and unknown in cell conditions) potency in PARP inhibition compared to Olaparib, additional pharmacological mechanisms of action may contribute to B-aza-steroid alkylators’ activity and potential to overcome the pharmacological resistance to current inhibitors.

## 5. Patents

This research is covered by the international patent WO2017001439.

## Figures and Tables

**Table 1:** Human ovarian cancer cell lines tested for the in vitro analysis of the compounds’ biological activity.

Cancer Type	Human Cell Line Designation	Mutated Genes	Special Characteristics
Ovarian Carcinoma	UWB1.289	p53	p53, cytokeratin 7 (CK-7) positive; calretinin
		(625delAG)	positive; Wilms’ tumor protein (WT) positive;
		BRCA1-null	Estrogen/Progesterone receptor negative;
		(2594delC)	MSS **
Ovarian Carcinoma	UWB1.289 + BRCA1	p53	Estrogen/Progesterone receptor negative; MSS **
		(625delAG)	
Ovarian Adenocarcinoma	SK-OV-3 (SKOV-3)	TP53 (p.S90fs)	Tumor Necrosis Factor; Diphtheria Toxin; Cisplatin and Adriamycin not sensitive; Estrogen receptor α (ERα) positive/Progesterone receptor negative; MSI-High *
		PIK3CA	
		(p.H1047R)	
Epithelial Ovarian Adenocarcinoma	NIH:OVCAR-3	MEK1	Androgen/Estrogen/Progesterone receptor positive; Cisplatin, Adriamycin not sensitive; Melphalan resistant; MSS **
		(p.G159R;	
		p.R160K;	
		p.A158) TP53	
		(p.R248Q)	

* MSI-high: High Microsatellite Instability, ** MSS: Microsatellite stable.

**Table 2:** In vitro cytostatic (IG50 and TGI ±SEM) (μM) and cytotoxic (IC50 ±SEM) (μM) effects of compounds ASA-A, ASA-B, ASA-C, POPA, 3-AB and Olaparib on all four studied human ovarian cancer cell lines in vitro. All values are representative of experiments performed in triplicate (Significance level, *p* < 0.01).

Human Cell Line	ASA-A			ASA-B		
	GI50	TGI	IC50	GI50	TGI	IC50
UWB1.289	19 ± 1.83	52 ± 4.61	71 ± 7.01	10 ± 0.81	35 ± 3.63	54 ± 5.11
UWB1.289 + BRCA1	22 ± 2.01	54 ± 4.33	76 ± 6.66	20 ± 1.86	72 ± 6.71	95 ± 9.03
SKOV-3	15 ± 1.26	33 ± 2.79	67 ± 7.11	24 ± 2.42	51 ± 4.46	98 ± 8.97
OVCAR-3	29 ± 3.04	38 ± 2.95	47 ± 4.82	44 ± 5.04	81 ± 7.82	>100
MRC-5	69 ± 7.13	>100	>100	86 ± 9.95	>100	>100
	**ASA-C**			**POPA**		
	**GI50**	**TGI**	**IC50**	**GI50**	**TGI**	**IC50**
UWB1.289	73 ± 7.58	>100	>100	64 ± 6.72	>100	>100
UWB1.289 + BRCA1	>100	>100	>100	>100	>100	>100
SKOV-3	>100	>100	>100	77 ± 7.54	>100	>100
OVCAR-3	>100	>100	>100	>100	>100	>100
MRC-5	>100	>100	>100	>100	>100	>100
	**3-AB**			**Olaparib**		
	**GI50**	**TGI**	**IC50**	**GI50**	**TGI**	**IC50**
UWB1.289	92 ± 12.98	>100	>100	14 ± 1.78	85 ± 9.34	>100
UWB1.289 + BRCA1	>100	>100	>100	>100	>100	>100
SKOV-3	>100	>100	>100	>100	>100	>100
OVCAR-3	>100	>100	>100	32 ± 4.64	>100	>100
MRC-5	>100	>100	>100	>100	>100	>100

**Table 3:** Michaelis Menten’s model kinetics data (± SEM) (of PARP (0.6 units/well) inhibition-induced by the 3-aminobenzamide (3-AB), B-lactam (dehydroepiandrosterone-derived) steroid alkylator ASA-A, B,D-bilactam (dehydroepiandrosterone-derived) steroid alkylator ASA-B, B-lactam (cholesten-derived) steroid alkylator ASA-C, alkylating component POPA and Olaparib.

Compounds	3-AB	ASA-A	ASA-B	ASA-C	POPA	Olaparib
Adj. R-Square	0.99449	0.93059	0.98691	0.98234	0.94566	0.97982
Vmax	74.130 ± 4.16	124.644 ± 60.83	147.817 ± 14.93	2.162 × 10^15^ ± 4.18 × 10^15^	310.114 ± 447.49	109.079 ± 10.23
Km	30.436 ± 4.43	111.851 ± 88.70	71.5031 ± 15.99	8.915 × 10^15^ ± 13.01 × 10^15^	676.425 ± 1227.41	1.117 ± 0.214
IC50 (μM)	63.06 ± 4.28	59.6 ± 6.42	35.16 ± 3.78	165.6 ± 19.77	132.68 ± 22.15	0.95 ± 0.12
IC100 (μM)	>150	>150	145.8 ± 14.29	>300	>300	13.17 ± 1.57

**Table 4:** Pearson’s correlation indexes and respective statistical significance levels (*p*) of cytostatic effects and PARP inhibition induced by the homo-aza lactamic steroid alkylators ASA-B, ASA-A, ASA-C and the alkylating acid POPA, as well as 3-AB on UWB1.289, UWB1.289 + BRCA1, OVCAR-3 and SKOV-3 human ovarian cancer cells.

		UWB1.289	UWB1.289 + BRCA1	OVCAR-3	SKOV-3
ASA-B	Pearson’s r	0.93	0.96	0.93	0.91
	Significance level (*p*)	<0.01	<0.01	<0.01	<0.01
ASA-A	Pearson’s r	0.95	0.97	0.91	0.90
	Significance level (*p*)	<0.01	<0.01	<0.01	<0.01
ASA-C	Pearson’s r	<0.25	<0.25	<0.25	<0.25
	Significance level (*p*)	NS *	NS	NS	NS
POPA	Pearson’s r	<0.50	<0.50	<0.50	<0.50
	Significance level (*p*)	NS	NS	NS	NS
3-AB	Pearson’s r	0.91	<0.50	<0.50	<0.50
	Significance level (*p*)	<0.01	NS	NS	NS

* NS: not significant (*p* > 0.05).

**Table 5:** Assessment of sister chromatid exchanges (SCEs) and respective PRIs induced in vitro by ASA-A, ASA-B, ASA-C, POPA, 3-AB and Olaparib at a drug concentration of 3 μM in PHA-stimulated PBMCs obtained from 5 healthy donors, as well as in UWB1.289 and UWB1.289 + BRCA1 human ovarian cancer cell metaphases. * Significance level *p* < 0.01, compared to control values; two-side paired *t*-test.

		**Control**	**ASA-A**	**ASA-B**	**ASA-C**	**POPA**	**3-AB**	**Olaparib**
Donor 1	SCEs/Metaphase ± SEM	8.23 ± 0.43	29.5 ± 1.4	36.5 ± 1.6	9.13 ± 0.45	8.4 ± 0.56	14.44 ± 0.92	20.43 ± 1.1
	SCE/Chromosome ± SEM	0.18 ± 0.01	0.64 ± 0.03	0.79 ± 0.03	0.2 ± 0.01	0.18 ± 0.01	0.31 ± 0.02	0.44 ± 0.02
	PRI	2.68	2.32	2.34	2.65	2.74	2.76	2.60
Donor 2	SCEs/Metaphase ± SEM	8.02 ± 0.46	30.7 ± 1.6	37.8 ± 1.9	8.46 ± 0.46	8.06 ± 0.43	13.26 ± 0.61	21.6 ± 1.4
	SCE/Chromosome ± SEM	0.17 ± 0.01	0.67 ± 0.03	0.82 ± 0.04	0.18 ± 0.01	0.18 ± 0.009	0.30 ± 0.013	0.47 ± 0.03
	PRI	2.72	2.26	2.29	2.74	2.78	2.70	2.56
Donor 3	SCEs/Metaphase ± SEM	8.43 ± 0.61	32.4 ± 1.7	41.7 ± 2.3	8.50 ± 0.55	8.54 ± 0.51	12.85 ± 0.73	23.87 ± 1.5
	SCE/Chromosome ± SEM	0.19 ± 0.013	0.70 ± 0.04	0.91 ± 0.05	0.18 ± 0.01	0.19 ± 0.011	0.28 ± 0.016	0.52 ± 0.03
	PRI	2.56	2.13	2.30	2.48	2.51	2.61	2.47
Donor 4	SCEs/Metaphase ± SEM	7.71 ± 0.35	27.2 ± 1.5	43.3 ± 2.4	8.61 ± 0.38	7.56 ± 0.31	13.21 ± 0.67	21.92 ± 1.2
	SCE/Chromosome ± SEM	0.17 ± 0.008	0.59 ± 0.03	0.94 ± 0.05	0.19 ± 0.01	0.16 ± 0.006	0.29 ± 0.015	0.48 ± 0.03
	PRI	2.84	2.21	2.18	2.72	2.73	2.86	2.76
Donor 5	SCEs/Metaphase ± SEM	9.12 ± 0.58	31.6 ± 1.7	38.4 ± 2.1	9.34 ± 0.64	9.02 ± 0.6	15.98 ± 0.96	25.32 ± 1.6
	SCE/Chromosome ± SEM	0.2 ± 0.012	0.69 ± 0.04	0.83 ± 0.05	0.2 ± 0.01	0.2 ± 0.013	0.35 ± 0.02	0.55 ± 0.04
	PRI	2.41	1.98	2.04	2.47	2.60	2.55	2.34
*Mean SCEs/metaphase Mean* *± SD*		*8.3* *± 0.53*	*30.28* ± 2.03*	*39.54* ± 2.84 *	*8.81* *± 0.4*	*8.32* *± 0.55*	*14.03* ± 1.24 *	*22.63* ± 1.95 *
*Mean SCE/Chromosome* *± SD*		*0.18* *± 0.013*	*0.66* ± 0.04 *	*0.86* ± 0.06 *	*0.19* *± 0.08*	*0.18* *± 0.011*	*0.30* ± 0.03 *	*0.66* ± 0.04 *
*Mean PRI* *± SD*		*2.642* *± 0.16*	*2.18* ± 0.13 *	2.23 ± 0.12 *	*2.612* *± 0.13*	*2.672* *± 0.11*	*2.696* *± 0.12*	*2.546* *± 0.16*
		**Control**	**ASA-A**	**ASA-B**	**ASA-C**	**POPA**	**3-AB**	**Olaparib**
UWB1.289	SCEs/Metaphase ± SEM	20.34 ± 1.68	73.78 ± 4.03 *	86.76 ± 4.97 *	24.53 ± 1.72	23.48 ± 1.58	31.91 ± 1.88 *	66.78 ± 4.45 *
	SCE/Chromosome ± SEM	0,27 ± 0.022	1.0 ± 0.054 *	1.17 ± 0.07 *	0.33 ± 0.02	0.32 ± 0.02	0.43 ± 0.025 *	0.90 ± 0.06 *
	PRI	2.18	1.88 *	1.77 *	2.15	2.22	2.14	1.98 *
UWB1.289 +BRCA1	SCEs/metaphase ± SEM	14.26 ± 1.14	63.86 ± 3.62 *	65.95 ± 3.47 *	15.32 ± 1.14	16.08 ± 1.26	14.86 ± 1.23	24.75 ± 1.86 *
	SCE/Chromosome ± SEM	0.19 ± 0.015	0.86 ± 0.05 *	0.89 ± 0.047 *	0.21 ± 0.02	0.22 ± 0.02	0.20 ± 0.017	0.33 ± 0.025 *
	PRI	2.09	1.85 *	1.88 *	2.08	2.11	2.16	2.07

## Data Availability

Data are available on request from the authors.
